# Analysis of autonomic function during natural defecation in patients with irritable bowel syndrome using real-time recording with a wearable device

**DOI:** 10.1371/journal.pone.0278922

**Published:** 2022-12-09

**Authors:** Rieko Nakata, Fumio Tanaka, Noriaki Sugawara, Yuichi Kojima, Toshihisa Takeuchi, Masatsugu Shiba, Kazuhide Higuchi, Yasuhiro Fujiwara

**Affiliations:** 1 Department of Gastroenterology, Osaka Metropolitan University Graduate School of Medicine, Osaka, Japan; 2 Second Department of Internal Medicine, Osaka Medical and Pharmaceutical University, Takatsuki City, Osaka, Japan; 3 Premier Departmental Research of Medicine, Osaka Medical and Pharmaceutical University, Takatsuki City, Osaka, Japan; School of Digestive & Liver Diseases, Institute of Post Graduate Medical Education & Research, INDIA

## Abstract

**Background:**

Autonomic dysfunction is a factor in irritable bowel syndrome (IBS). However, there are no reports of autonomic nervous system (ANS) activity during natural defecation in patients with IBS. We aimed to clarify the relationship between ANS activity and life events, such as defecation and abdominal symptoms, using real-time recording.

**Methods:**

Six patients with IBS and 14 healthy controls were enrolled in this prospective multicenter study. ANS activity was recorded for 24 h using a T-shirt wearable device, and life events were recorded simultaneously in real time using a smartphone application software. Low frequency/high frequency (LF/HF) and HF calculated by power spectrum analysis were defined as activity indicators of the sympathetic and parasympathetic nerves, respectively.

**Results:**

The means of LF/HF and HF in the period with positive symptoms were comparable between the groups; however, the sum of LF/HF, sum of ΔLF/HF, and the maximum variation in ΔLF/HF were significantly higher in the IBS group. In the IBS group, the sum of ΔLF/HF and LF/HF increased significantly from 2 min before defecation, and the sum of LF/HF remained significantly higher until 9 min after defecation. The sum of ΔLF/HF at 2 min before defecation was significantly positively correlated with the intensity of abdominal pain and diarrhea and constipation scores. In contrast, it was significantly negatively correlated with defecation satisfaction and health-related quality of life.

**Conclusions:**

In patients with IBS, sympathetic nerve activity was activated 2 min before defecation, which was correlated with abdominal symptoms and lower QOL.

## Introduction

Irritable bowel syndrome (IBS) is a chronic gastrointestinal disorder characterized by abdominal pain associated with defecation and altered bowel habits. The prevalence of IBS is approximately 10%, and its rate is higher in younger people than in older adults [1]. The pathophysiology of IBS is presumed to involve gastrointestinal motility, visceral hypersensitivity, genetic and immunological factors, increased intestinal permeability, altered gut microbiota, dietary factors, and psychosocial factors [2]. Multiple factors are thought to contribute to IBS, and gut-brain interactions are particularly important concepts that involve these factors. Gut-brain interactions are involved in the gut-associated immune system, enteric nervous system, neuro-hormonal, and gut microbiome [3, 4]. The autonomic nervous system (ANS) is an important factor in gut-brain interactions [5].

A common measure used to assess ANS activity is heart rate variability (HRV), which has been used in many studies on IBS [6]. Electrocardiograms (ECGs) are used to evaluate HRV, and the median frequency domain indices of HRV across 24 h have been reported to show increased sympathetic nerve activity and decreased parasympathetic nerve activity in patients with IBS [7]. Moreover, it has been reported that sympathetic nerves dominate in patients with IBS for 30 min after food intake [8] and during rapid eye movement sleep [9], indicating autonomic dysfunction. Several reports have evaluated changes in ANS activity in the sitting and supine positions as well as in deep breathing [10, 11].

Moreover, previous studies have attempted to evaluate ANS activity during colorectal distention using balloon inflation as a model for evaluating pain perception and visceral hypersensitivity, although the results obtained thus far are controversial [12–15]. Most previous reports have shown that the parasympathetic nerve activity is decreased due to colorectal distension in IBS, while the changes in sympathetic nerve activity differ in each report. This model is useful for evaluating visceral hypersensitivity; however, it also has some limitations. Because balloon inflation is invasive and not a physiological condition, participants may experience psychological and physiological stress. Therefore, the results of these studies may be affected by the stress conditions. Furthermore, this model cannot be used to evaluate ANS activity during natural defecation.

IBS was defined on the basis of the correlation between abdominal pain and defecation. Therefore, it is important to evaluate ANS activity during natural defecation to better understand IBS pathophysiology. However, there have been no reports on ANS activity during natural defecation and its correlation with abdominal symptoms in patients with IBS because it is technically difficult to perform ECG and symptom assessment during defecation. Accordingly, we conducted real-time recordings of ANS activity during natural defecation and abdominal symptoms using wearable devices. The advantage of real-time recording is that objective and subjective data can be simultaneously obtained. The aim of this study was to clarify the association between ANS activity and life events, including defecation and abdominal symptoms, using real-time recording in IBS patients. We obtained information on ANS activity with a T-shirt-type wearable device and developed a smartphone application software for real-time recording of life events and abdominal symptoms.

## Materials and methods

### Subjects

This was a prospective multicenter study in which patients with IBS visiting Osaka City University and Osaka Medical and Pharmaceutical University participated between July 2018 and May 2019. All patients with IBS met the Rome IV criteria and had received a medical diagnosis of IBS prior to the study. In the control group, sex- and age-matched subjects were recruited via the Internet; they did not have gastrointestinal symptoms and did not meet the Rome IV criteria for IBS and functional dyspepsia. Participants in both groups were excluded if they had a history of gastrointestinal surgery or used a pacemaker or an implantable cardioverter defibrillator. We compared the data obtained between patients with IBS and controls. The study protocol was approved by the Ethics Committee of the Osaka City University Graduate School of Medicine (No. 3686; approved on January 25, 2017). Written informed consent was obtained from all of the participants in this study. If consent was waived, patients were not included in the study.

### Study protocol

**Fig 1:**
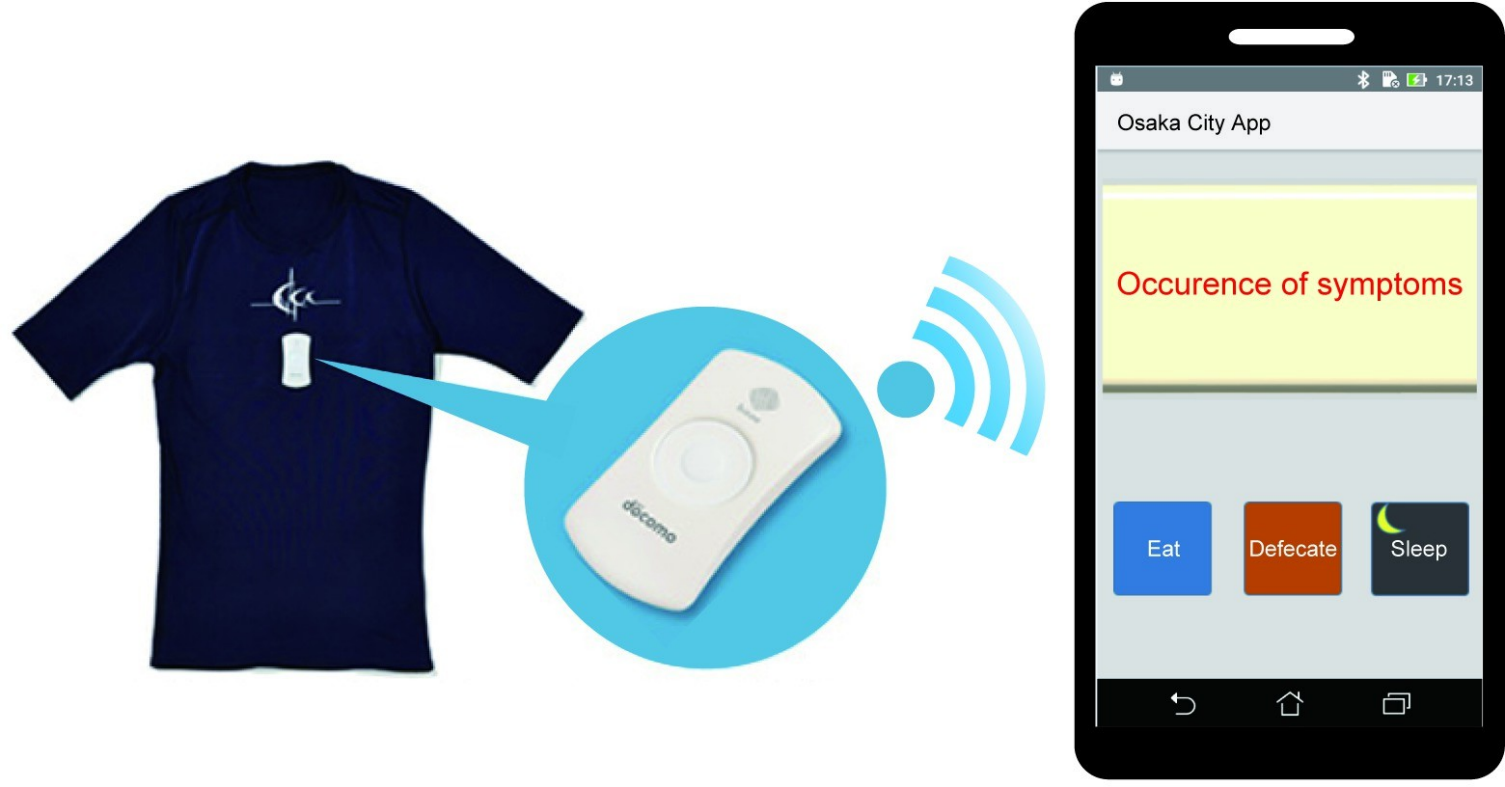
T-shirt-type wearable device and smartphone application software. Heart rate variability (HRV) was measured using a T-shirt wearable device attached to a transmitter in front of the T-shirt. The HRV data were transferred and recorded on a smartphone using Bluetooth. At the same time, life events, such as abdominal symptoms, defecation, eating, and awakening or sleep, were recorded in real time using a smartphone application software during HRV recording.

HRV was recorded using a Hitoe® (TORAY Industries, Inc., Tokyo, Japan; NTT DOCOMO, Inc., Tokyo, Japan), which is a wearable T-shirt device, to monitor the 24-h HRV every 40 millisecond (ms) (Fig 1). This device has been used as a tool for measuring ANS activity in sports medicine and post-stroke patients, and its validity has been demonstrated [16, 17]. Simultaneously, life events, such as the timing of the appearance or disappearance of abdominal symptoms, defecation, eating, and awakening or sleep, were recorded in real time using a smartphone application software during HRV recording. This software was newly developed in a joint effort by NTT DATA KANSAI Corporation, NTT DATA MSE Corporation, Osaka City University, and Osaka Medical and Pharmaceutical University and is not sold in the open market. The participants conducted normal daily activities until the 24-h period was over.

In addition, all participants were asked to complete questionnaires including the IBS severity scoring system (IBS-SSS) [18], the gastrointestinal symptom rating scale (GSRS) [19], the Hospital Anxiety and Depression Scale (HADS) [20], and the Japanese version of the 8-item Short-Form Health Survey (SF-8) [21] prior to measuring ANS activity. The SF-8 measures health-related quality of life (QOL) based on scores from eight domains and two summaries, including physical functioning, physical role, bodily pain, general health perception, vitality, social functioning, emotional role, mental health, and physical and mental component summaries, with lower scores reflecting a worse QOL.

### Evaluation of ANS

HRV data obtained from the Hitoe® were analyzed by RR interval power spectral analysis using software developed at the same time as the smartphone application software. We performed power spectrum analysis as in previous studies [10, 22] and classified the results into three frequency components: very low frequency power (0.007–0.035 Hz), low frequency power (LF) (0.035–0.15 Hz), and high frequency power (HF) (0.15–0.5 Hz). The ratio of LF to HF (LF/HF) and HF were used as indicators of sympathetic and parasympathetic nervous system activities, respectively.

**Fig 2:**
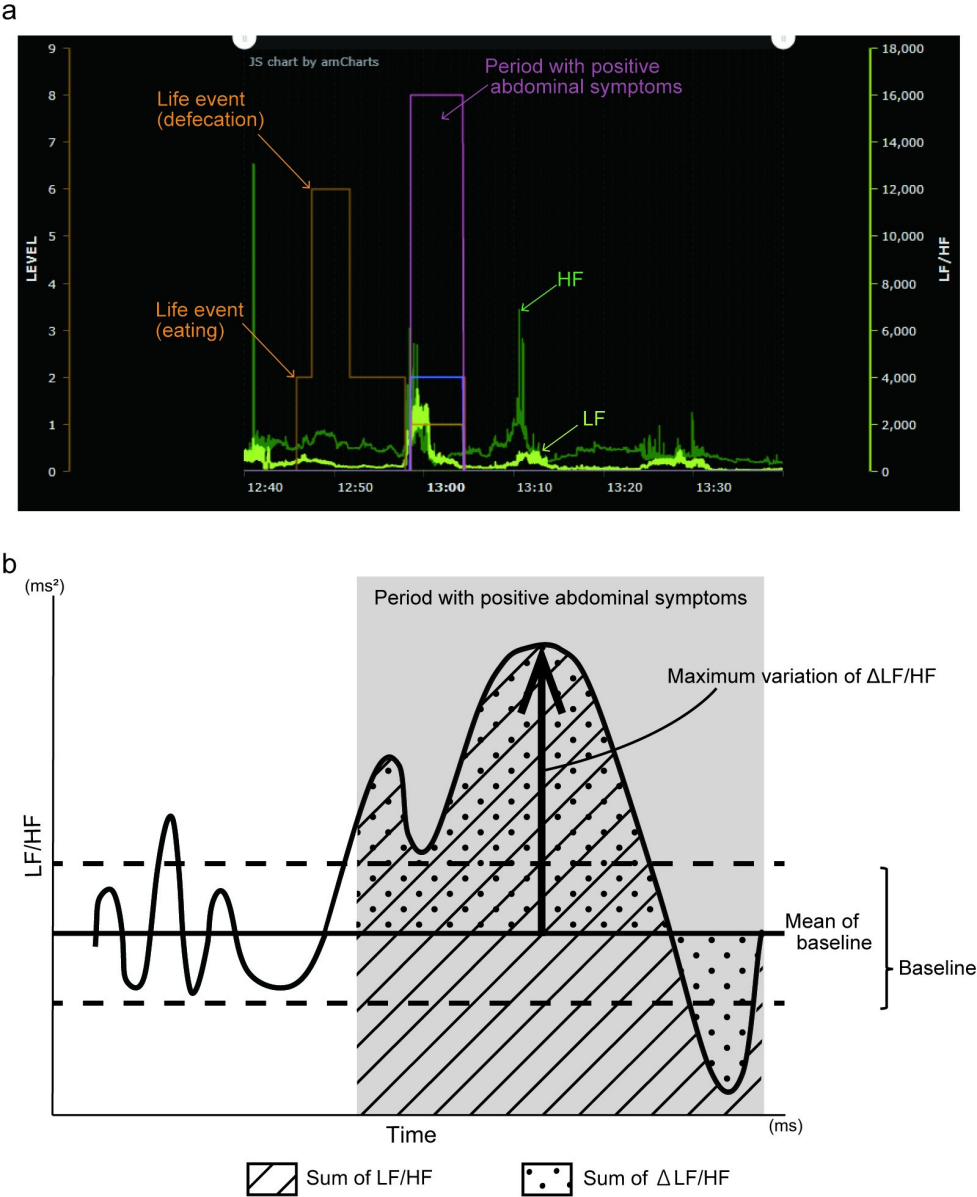
The graph of obtained data and measuring method of ANS activity. (a) The real graph of obtained data about autonomic nervous system (ANS) activity and life events. The results of low frequency (LF) (yellow line) and high frequency (HF) (green line) represented in a graph. Life events, such as defecation and eating (the enclosed area with orange line) and period with positive symptoms (the enclosed area with pink line), were recorded. (b) Measuring method of ANS activity. The baseline LF/HF was defined as the value included in the range of the mean ± 2 standard deviations (dashed line) of LF/HF measured in the period with no symptoms. The sum of LF/HF was the area under the curve of LF/HF measured in the period with positive symptoms (upward diagonal). The sum of ΔLF/HF was the sum of variation from the mean value of baseline measured in the period with positive symptoms (dots). The maximum variation of ΔLF/HF indicated the maximum variation from the mean value of baseline measured in the period with positive symptoms (arrow). HF was analyzed in the same way.

A real graph of the obtained data on ANS activity and life events is shown in Fig 2A. The baseline LF/HF ratio was defined as the value included in the range of the mean ± 2 standard deviations of LF/HF measured during periods with no symptoms (Fig 2B). The sum of LF/HF was calculated as the area under the curve of LF/HF measured during periods with positive symptoms. The sum of ΔLF/HF was calculated as the sum of the variation from the baseline LF/HF measured during periods with positive symptoms. The maximum variation in ΔLF/HF indicated the maximum variation from the baseline of LF/HF measured during periods with positive symptoms. The definition of an abnormal LF/HF ratio was outside the baseline range. The HF was analyzed in the same way. Moreover, inter-beat-interval (IBI) measures were calculated from the raw ECG signal. The mean IBI and standard deviation of the normal-to-normal interval (SDNN) were derived from IBI data. The coefficient of variation of RR intervals (CVRR) was represented as the ratio of SDNN to mean IBI.

The main outcome was changes in the autonomic nervous function before and after defecation, which were compared between the control and IBS groups. Other measured values of autonomic nervous function were compared as secondary outcomes. Furthermore, correlations between the measured values of autonomic nervous function, symptom scores, and QOL scores were evaluated.

### Statistical analysis

Data are expressed as medians and interquartile ranges for continuous variables and as numbers for categorical variables. Continuous variables of the two groups were compared using the Mann–Whitney U test. Categorical variables were compared using Fisher’s exact test. Correlations between the measured values of autonomic nervous function and symptom scores or QOL scores were obtained using Spearman correlation analysis.

## Result

### Patient characteristics

In this study, HRV was measured in 7 patients with IBS based on the Rome IV criteria and 23 healthy controls. Among the participants, in 2 controls, we could not record the HRV for > 10 h, and one patient and nine controls were excluded as they did not defecate during the recording. Finally, we compared the data of six patients with IBS to 14 controls. As shown in Table 1, the participants in each group had similar age, sex ratio, and body mass index. The prevalence of diarrhea-predominant IBS was 83.3% in patients with IBS. The frequency and intensity of abdominal pain and intensity of abdominal distension in the IBS group were significantly higher than those in the control group. In addition, the levels of satisfaction with defecation were lower in the IBS group. The total GSRS score was significantly higher in the IBS group than in the control group, especially for diarrhea and constipation. The anxiety and depression scores did not differ significantly between the two groups. In the SF-8, the components of role physical, social functioning, and physical component summary-8 were significantly lower in the IBS group, which indicated lower health-related QOL.

**Table 1:** Background characteristics.

	Controls (n = 14)	IBS (n = 6)	*p*–value
Age, years	51.0 (42.5–55.0)	50.5 (39.8–65.8)	0.84
Male, n (%)	7 (50.0)	3 (50.0)	1.00
BMI, kg/m^2^	22.3 (21.0–23.3)	21.4 (20.5–22.1)	0.25
Sleep period, h	6.3 (6.0–7.0)	6.0 (6.0–6.8)	0.86
Frequency of defecation, times/day	1.0 (1.0–1.8)	2.8 (1.8–3.8)	0.01
Subtype of irritable bowel syndrome			
Diarrhea, n (%)	0 (0.0)	5 (83.3)	< 0.01
Constipation, n (%)	0 (0.0)	1 (16.7)	0.30
Medication for IBS			
Ramosetron, n (%)	0 (0.0)	2 (33.3)	0.08
Polycarbophil, n (%)	0 (0.0)	1 (16.7)	0.30
Frequency of abdominal pain, point	0 (0–0)	3.5 (2.3–4.8)	< 0.01
Intensity of abdominal pain, point	0 (0–0)	25.0 (5.0–45.0)	< 0.01
Intensity of abdominal distension, point	0 (0–0)	25.0 (6.3–43.8)	0.01
Satisfaction levels of defecation, %	70.0 (70.0–88.8)	30.0 (30.0–30.0)	0.01
GSRS			
Total score	16.5 (15.0–21.0)	35.0 (32.0–38.8)	< 0.01
Abdominal pain score	3.0 (3.0–3.0)	3.5 (3.0–4.0)	0.20
Diarrhea score	3.0 (3.0–5.5)	10.5 (8.5–14.0)	< 0.01
Constipation score	3.0 (3.0–4.0)	6.5 (5.3–7.8)	0.01
SF-8			
General health perception	50.3 (50.3–50.3)	45.3 (40.4–50.3)	0.15
Physical functioning	53.5 (53.5–53.5)	53.5 (44.5–53.5)	0.11
Role physical	54.1 (54.1–54.1)	44.0 (40.7–52.4)	< 0.01
Bodily pain	60.4 (52.5–60.4)	49.3 (46.1–58.4)	0.13
Vitality	53.7 (46.8–53.7)	44.5 (40.0–51.4)	0.05
Social functioning	55.1 (55.1–55.1)	50.4 (45.6–55.1)	0.03
Mental health	50.7 (46.4–55.4)	47.8 (44.9–50.7)	0.23
Role emotional	54.2 (48.0–54.2)	48.0 (43.7–52.7)	0.17
Physical component summary-8	48.3 (47.0–51.0)	43.6 (36.9–46.7)	0.01
Mental component summary-8	51.2 (46.0–53.5)	47.2 (44.7–49.5)	0.28
HADS			
Anxiety score	2.0 (1.0–4.0)	3.5 (2.3–6.3)	0.23
Depression score	1.5 (0–4.8)	4.5 (2.5–6.5)	0.13

Data are expressed as median (interquartile range) for continuous variables and as numbers (percentage) for categorical variables.

IBS: Irritable bowel syndrome, BMI: Body mass index, GSRS: Gastrointestinal symptom rating scale, SF-8: The Japanese version of the 8-item Short-Form Health Survey, HADS: Hospital Anxiety and Depression Scale

### Measurement data of ANS activity

There were no significant differences in the means of LF/HF and HF between the two groups during any period: baseline (asymptomatic), awake period, and sleep period (Table 2). In periods with positive symptoms, the total number of ANS signals was significantly higher in the IBS group, indicating that the period with positive symptoms was longer in the IBS group. In periods with positive symptoms, the sum of LF/HF, sum of ΔLF/HF, and maximum variation of ΔLF/HF in the IBS group were significantly higher than those in the control group, although the mean LF/HF was not significantly different. These results indicate increased sympathetic activity only during the period with positive IBS symptoms. Moreover, the sum of HF, sum of ΔHF, and maximum variation of ΔHF were significantly higher in the IBS group than those in the control group. These results indicate that parasympathetic activity also increased in periods with positive symptoms. The number of abnormal LF/HF ratio was significantly higher in the IBS group. In contrast, the number of abnormal HF was not statistically different between the 2 groups. There were no significant differences in CVRR (*p* = 0.32; 15.6 [14.6–19.0] vs. 19.7 [16.0–24.1]), mean IBI (*p* = 0.93; 874 [738–991] vs. 836 [791–877]), and SDNN (*p* = 0.32; 152 [114–181] vs. 172 [140–190]) between the control group and the IBS group.

**Table 2:** Autonomic nervous system activity in healthy controls and patients with IBS.

	Controls (n = 14)		IBS (n = 6)		
	Median	IQR	Median	IQR	*p*–value
All period					
Mean of LF/HF, ms^2^	3.7	2.5–4.0	3.3	2.0–4.1	0.49
Mean of HF, ms^2^	154.1	129.5–208.9	273.9	156.5–432.2	0.35
Total power, ms^2^	1818.2	1631.7–2660.4	2303.3	1668.1–4183.5	0.60
Baseline					
Mean of LF/HF, ms^2^	3.2	2.1–3.6	3.1	1.7–3.7	0.49
Mean of HF, ms^2^	143.4	111.7–166.6	256.7	125.9–398.7	0.21
Awake period					
Mean of LF/HF, ms^2^	2.9	2.5–3.1	2.8	1.7–3.5	0.78
Mean of HF, ms^2^	163.8	135.6–350.2	374.2	180.4–562.9	0.31
Sleep period					
Mean of LF/HF, ms^2^	3.9	2.3–4.7	3.7	2.3–4.1	0.40
Mean of HF, ms^2^	129.2	94.4–158.1	157.2	97.2–297.2	0.72
Period with positive symptoms					
Total number of ANS signals	4.0	0–125.0	1901.0	843.5–3841.5	0.01
Mean of LF/HF, ms^2^	2.3	0–4.0	2.9	2.5–3.8	0.27
Sum of LF/HF, ms^2^	13.0	0–2023.8	6014.6	3804.4–10527.3	0.01
Sum of ΔLF/HF, ms^2^	1.2	0–347.7	1994.3	1397.1–4806.2	0.01
Maximum variation of ΔLF/HF, ms^2^	0.7	0–8.2	7.2	4.9–500.7	0.04
Mean of HF, ms^2^	29.3	0–205.8	145.4	114.1–337.0	0.08
Sum of HF, ms^2^	761.6	0–40640.4	178696.5	148067.3–1103482.0	0.01
Sum of ΔHF, ms^2^	77.3	0–7784.1	154203.9	45418.9–707833.4	0.01
Maximum variation of ΔHF, ms^2^	47.7	0–345.3	8336.1	520.2–65439.1	0.02
Number of abnormal LF/HF	0	0–0	6.5	5.3–30.3	0.01
Number of abnormal HF	0	0–3.8	2.0	0–117.3	0.41

IBS: Irritable bowel syndrome, IQR: Interquartile range, LF: Low frequency power, HF: High frequency power, ANS: Autonomic nervous system

### Changes in autonomic nervous function before and after defecation

**Fig 3:**
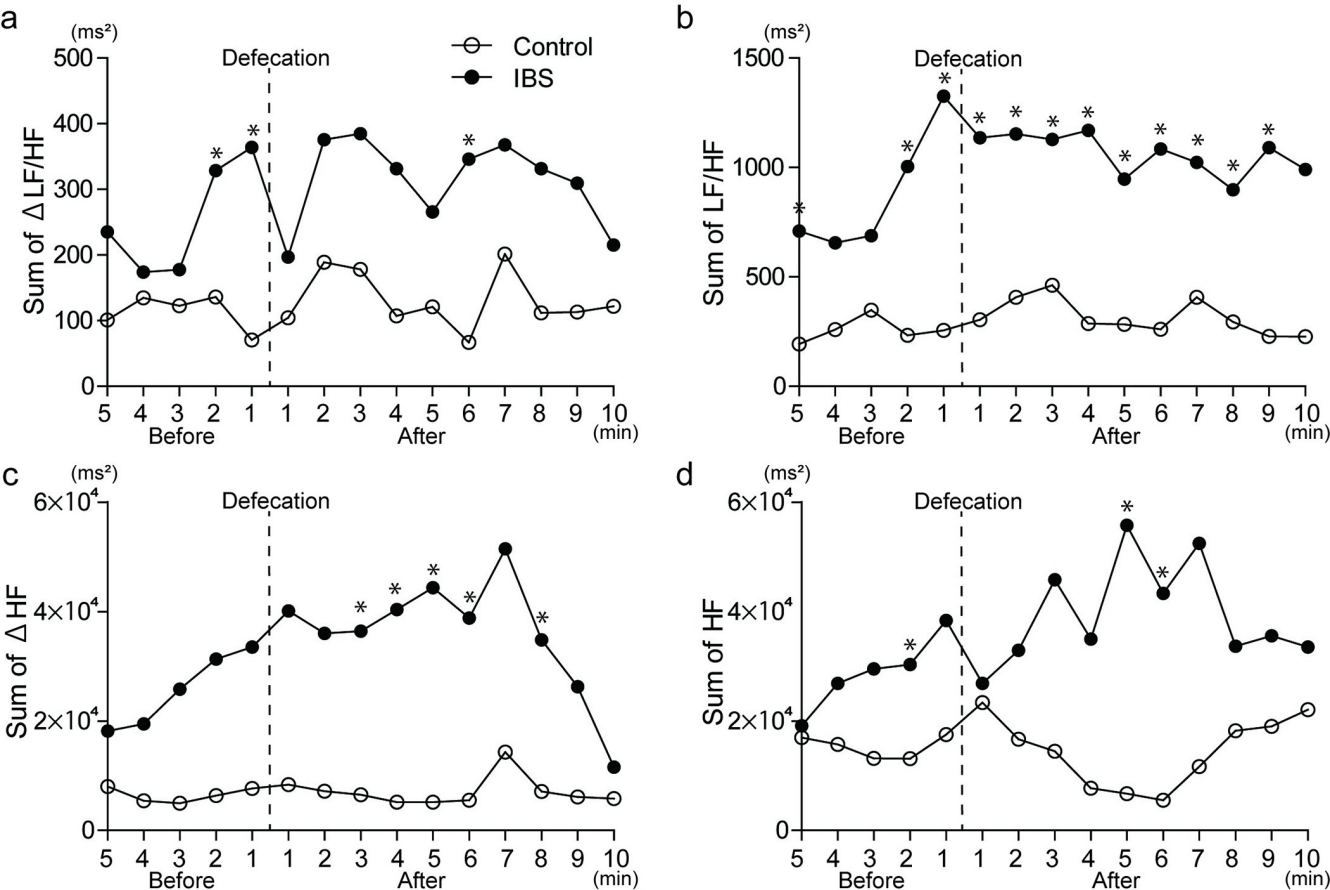
Autonomic nervous activities before and after defecation. (a, b) Changes in sympathetic nerve activities before and after defecation were evaluated by the sum of Δlow frequency/high frequency (LF/HF) and the sum of LF/HF, respectively. (c, d) Changes in parasympathetic nerve activities before and after defecation were evaluated by the sum of ΔHF and the sum of HF, respectively. Data represents the median. Statistical significance was calculated by Mann–Whitney test (*p < 0.05).

The sum of ΔLF/HF and LF/HF increased significantly from 2 min before defecation in the IBS group compared to that in the control group (Fig 3A and 3B). In addition, the sum of the LF/HF remained significantly higher until 9 min after defecation (Fig 3B). A similar tendency was observed in the sum of ΔLF/HF, although the values were not statistically significant, except at 6 min after defecation (Fig 3A). In contrast, the sum of ΔHF increased significantly from 3 to 8 min after defecation in the IBS group (Fig 3C). A similar tendency was observed for the sum of HF, which was significantly higher from 5 to 6 min after defecation in the IBS group (Fig 3D).

### Correlation among the sum of ΔLF/HF at 2 min before defecation and symptom scores and QOL scores

**Fig 4:**
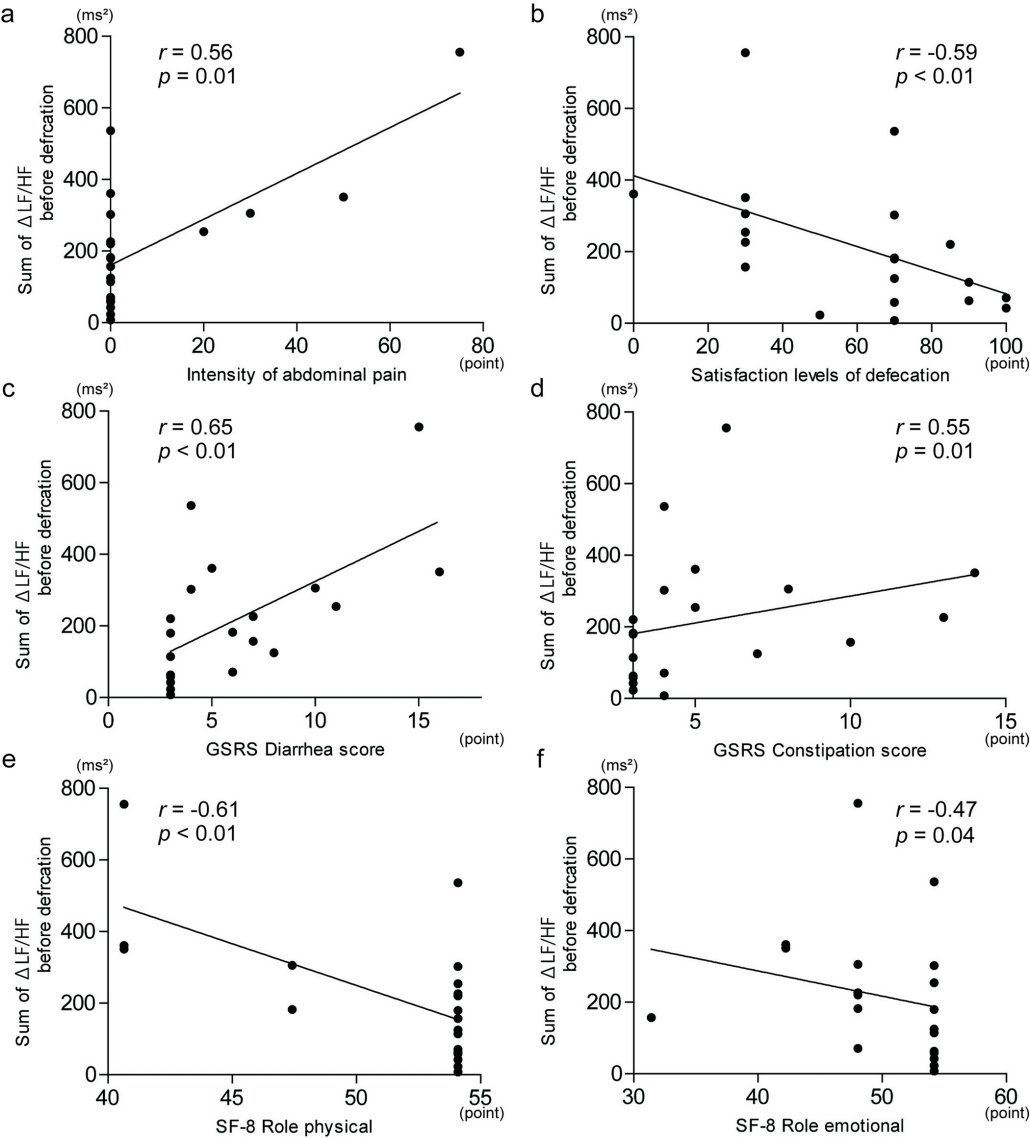
Relationship among sympathetic nerve activity at 2 min before defecation, symptoms, and quality of life. Sympathetic nerve activity was evaluated by the sum of Δlow frequency/high frequency (LF/HF). (a) Intensity of abdominal pain, (b) satisfaction levels of defecation, (c) gastrointestinal symptom rating scale (GSRS) diarrhea score, (d) GSRS constipation score, (e) the Japanese version of the 8-item Short-Form Health Survey (SF-8) role physical, and (f) the SF-8 role emotional score were significantly correlated with the sum of ΔLF/HF at 2 min before defecation. Data were analyzed by Spearman test.

The sum of ΔLF/HF at 2 min before defecation correlated positively with the intensity of abdominal pain (Fig 4A; *r* = 0.56, *p* = 0.01), whereas it was negatively correlated with satisfaction levels of defecation (Fig 4B; *r* = –0.59, *p* < 0.01). The sum of ΔLF/HF at 2 min before defecation had statistically significant positive correlations with the diarrhea score (Fig 4C; *r* = 0.65, *p* < 0.01) and constipation score in the GSRS (Fig 4D; *r* = 0.55, *p* = 0.01). In addition, the sum of ΔLF/HF at 2 min before defecation had statistically significant negative correlations with the values of role physical (Fig 4E; *r* = –0.61, *p* < 0.01) and role emotional in the SF-8 (Fig 4F; *r* = –0.47, *p* = 0.04).

## Discussion

This study showed that in patients with IBS, the sympathetic nerve was activated from 2 min before defecation, and the activity continued until 9 min after defecation. In addition, sympathetic nerve activation at 2 min before defecation was associated with the intensity of abdominal pain and QOL. This is the first study to evaluate ANS activity before and after defecation with abdominal pain using a newly developed real-time recording device under natural physiological conditions.

Neurons of the central nucleus of the amygdala are activated by exposure to stress, such as pain and discomfort [23]. Projections from the central nucleus of the amygdala to the lateral hypothalamus contribute to the arousal of the ANS [24]. Projections of peptidergic neurons in the hypothalamic paraventricular nucleus secrete corticotrophin-releasing hormone (CRH), which acts on the anterior pituitary gland to release adrenocorticotrophic hormone, into the primary plexus of blood vessels, which comprise the hypothalamo-hypophyseal portal system. Adrenocorticotrophic hormone stimulates the production and release of cortisol from the adrenal cortex [25]. Several studies have reported that CRH and cortisol are involved in the stress response [26, 27]. Thus, these responses lead to the “fight or flight” response under stress.

**Fig 5:**
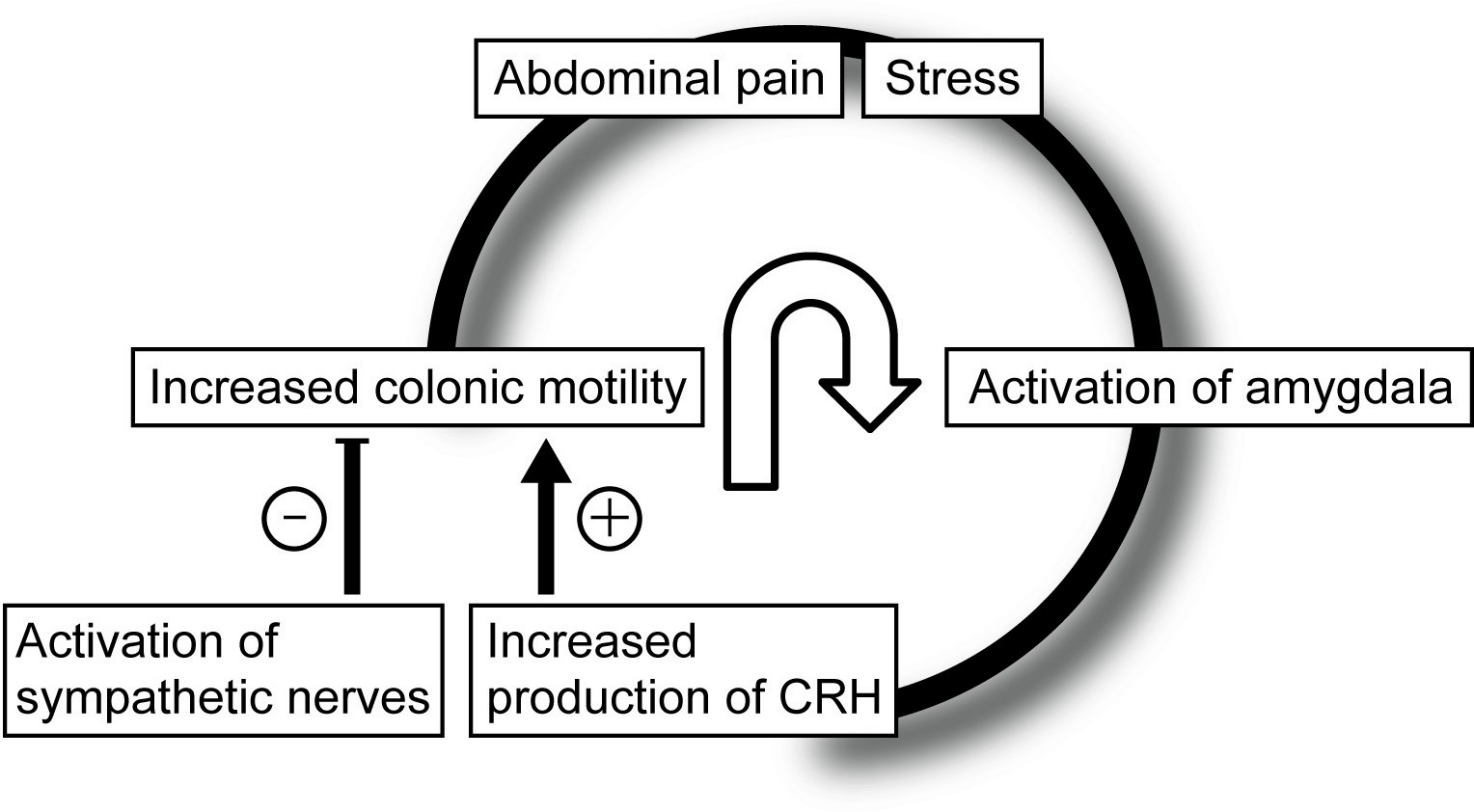
Hypothesized mechanism of exacerbated symptoms and increased sympathetic nerve activity before defecation. The stress of abdominal pain activates the amygdala to release corticotrophin-releasing hormone (CRH), resulting in further exacerbation of abdominal pain by increasing colonic motility. These cascades “exacerbate the circle of pain.” Two minutes before defecation, the stress also activates sympathetic nerves as a stress response against abdominal pain. Increased sympathetic nerve activity can decrease colonic motility, which may have a protective role to hold back the defecation. This situation may be under competition between the accelerator of colonic motility caused by CRH and the brake caused by sympathetic nerve activation.

A meta-analysis demonstrated that in response to rectal inflation, the amygdala was activated to a greater extent in patients with IBS than in healthy controls [28]. It has been reported that intravenous administration of CRH increases the motility of the descending colon and prolongs the time with abdominal symptoms in patients with IBS [29]. Additionally, exogenous CRH induced greater activation in the amygdala of patients with IBS than in controls [30]. Considering these facts, stress caused by abdominal pain leads to activation of the amygdala and CRH secretion as a stress response, resulting in increased colonic motility. Subsequent to the increased motility following stimulation via the colonic nociceptor, further abdominal pain occurs, leading to an “exacerbating circle of pain” (Fig 5). In contrast, it is known that sympathetic nerve activation as a result of the stress response exerts an inhibitory effect on colonic motility [31]. In this study, sympathetic nerve activation 1 to 2 min before defecation was considered to reflect the stress response to abdominal pain because it was positively correlated with the intensity of abdominal pain and negatively correlated with health-related QOL. In addition, it is presumed that colonic motility 1 to 2 min before defecation is in a state of holding back the defecation because this situation may be under competition between the accelerator of colonic motility caused by CRH and the brake caused by the activation of sympathetic nerves.

In our study, the sum of LF/HF was significantly higher in the IBS group for 9 min after defecation, indicating that the activation of sympathetic nerves continued even when patients with IBS finished defecation. This activation was supposed to reflect the sustained stress after defecation, which disappeared 9 min after defecation. These results are congruent with previous reports that have shown that defecation does not improve abdominal pain in a large number of patients with IBS, but rather aggravates it [32]. In contrast, the sum of ΔHF was significantly higher in the IBS group after defecation, indicating that the parasympathetic nerves were more active after defecation than before. The parasympathetic nerve was most activated in the delayed time phase of 3 to 8 min after defecation, compared to the time at which the sympathetic nerve was most activated around 2 min before defecation. In other words, there is a gap in the time phase of activation between the sympathetic and parasympathetic nerves. First, the sympathetic nerve was activated before defecation and then the parasympathetic nerve was activated before defecation. The activity of the parasympathetic nerve is considered a compensatory reaction that normalizes the balance of the autonomic nerve, in which the sympathetic nerve is predominantly activated during defecation.

Our study has two critical strengths. First, we could perform real-time recording of life events and ANS activities using a wearable device and newly developed smartphone application software. Previous studies have only evaluated ANS activity during visceral pain perception by increasing colonic pressure using balloon inflation or sigmoidoscopy. The limitation of previous methods is that they are artificial conditions and could not evaluate ANS activity under physiological conditions of natural defecation with abdominal pain. As a strength of our study, we could evaluate ANS activity under natural conditions by simultaneously measuring HRV, life events, and symptoms. Real-time recording enables an accurate evaluation of the association between them. Second, as HRV was measured with a T-shirt-type wearable device, subjects felt less uncomfortable during the measurement. In most previous studies evaluating the ANS in patients with IBS, an ECG and a device that attaches a cord to the chest were used, which were considered to increase stress. Compared to healthy subjects, patients with IBS show exacerbated symptoms [33] and an increased stress response [34] due to added stress; therefore, it is important to reduce discomfort during measurement to accurately evaluate the ANS.

The present study has some limitations. First, the sample size was small because 10 subjects who did not defecate during recording were excluded. Therefore, the ability to detect changes in ANS activity between patients with IBS and controls may be decreased, and the results of this study may be different in a large cohort. Second, the connection of the wearable device was sometimes poor owing to the design issues of the device, resulting in a partial lack of recording during measurement. Third, because the actions of the participants, such as eating during periods with positive symptoms, were not restricted during the measurements, it was assumed that there was variability in the measured data. However, to measure the actual autonomic function of patients with IBS, it was necessary to measure it during their usual activities. Finally, in the IBS group, patients who had improved abdominal symptoms due to medication were included. Therefore, it is possible that ANS activity was partly normalized by medication. Further studies are needed to increase the sample size and include only patients without medication among patients with IBS.

In conclusion, our results provide new and important findings that sympathetic nerves are activated 2 min before defecation in patients with IBS, which is associated with the intensity of abdominal pain and lower QOL. These results may open a new field for understanding the pathophysiology of IBS and enable the development of a therapeutic approach to ANS activity during defecation.

## Supporting information

S1 File Data sheet of study participants. (CSV)
